# Associated factors for recommending HBV vaccination to children among Georgian health care workers

**DOI:** 10.1186/1471-2334-12-362

**Published:** 2012-12-20

**Authors:** Maia Butsashvili, George Kamkamidze, Marina Topuridze, Dale Morse, Wayne Triner, Jack DeHovitz, Kenrad Nelson, Louise-Anne McNutt

**Affiliations:** 1 National Center for Disease Control and Public Health (NCDC), 9 Asatiani st., Tbilisi, 0177, Georgia; 2 Maternal and Child Care Union, Tbilisi, Georgia; 3 Division of Foodborne, Waterborne and Environmental Diseases at CDC, Atlanta, GA, USA; 4 Department of Emergency Medicine, at Albany Medical College, Albany, NY, USA; 5 Department of Medicine, AT SUNY-Downstate Medical Center, Brooklyn, NY, USA; 6 Johns Hopkins Bloomberg School of Public Health, Baltimore, MD, USA; 7 University at Albany, School of Public Health, Rensselaer, NY, USA

**Keywords:** Hepatitis B, Vaccine, Safety, Health Care Worker, Newborns

## Abstract

**Background:**

Most cases of hepatitis B virus (HBV) infection and subsequent liver diseases can be prevented with universal newborn HBV vaccination. The attitudes of health care workers about HBV vaccination and their willingness to recommend vaccine have been shown to impact HBV vaccination coverage and the prevention of vertical transmission of HBV. The purpose of this study was to ascertain the factors associated with health care worker recommendations regarding newborn HBV vaccination.

**Methods:**

A cross-sectional study of prevalence and awareness of hepatitis B and hepatitis B vaccine was conducted among randomly selected physicians and nurses employed in seven hospitals in Georgia in 2006 and 2007. Self-administered questionnaires included a module on recommendations for HBV, HCV and HIV.

**Results:**

Of the 1328 participants included in this analysis, 36% reported recommending against hepatitis B vaccination for children, including 33% of paediatricians. Among the 70.6% who provided a reason for not recommending HBV vaccine, the most common concern was an adverse vaccine event. Unvaccinated physicians and nurses were more likely to recommend against HBV vaccine (40.4% vs 11.4%, PR 3.54; 95% CI: 2.38, 5.29). Additionally, health care worker age was inversely correlated with recommendations for HBV vaccine with older workers less likely to recommend it.

**Conclusion:**

Vaccinating health care workers against HBV may provide a dual benefit by boosting occupational safety as well as strengthening universal coverage programs for newborns.

## Background

Hepatitis B virus (HBV) infection remains a major global public health concern. Georgia is among the countries with an intermediate prevalence of chronic HBV infection (2.7%), similar to that of other Eastern European and Central Asian countries [1-3]. The risk of hepatitis due to vertical transmission or from exposure in early childhood (e.g., medical exposure to contaminated equipment, household transmission, child-to-child transmission) is of particular concern because infection at a young age is associated with a substantial increased risk of liver disease (e.g., cirrhosis, hepatocellular carcinoma) later in life [4,5].

Fortunately, HBV is preventable through hepatitis B vaccination. The vaccine has been shown to be safe and efficacious for newborns and children [6,7]. Countries which have implemented universal newborn HBV vaccination have reported substantial declines in HBV incidence that is associated with these increases in vaccination coverage [8,9]. Thus, most cases of HBV and its sequelae could be prevented by a universal newborn HBV vaccination program. The success of such public health initiatives depends largely on the degree to which health care providers promote vaccination.

Despite the availability of free vaccine for newborns in Georgia, anecdotal information suggested that health care worker (HCW) concerns about the safety of the HBV vaccine posed an important challenge to universal vaccine coverage for newborns [10]. The study conducted among 297 HCWs reported that only 54% of study participants would recommend the vaccine to other HCWs. Perception of vaccine safety was identified as the most important predictor for acceptance (Prevalence Ratio (PR)=3.25 [95% CI:1.19, 8.90]) and for willingness to recommend HBV vaccine to their colleagues [11].

While neonatologists and pediatricians are responsible for newborn HBV vaccinations, HCWs of other specialties likely have a significant indirect impact on vaccine coverage in the general population. Georgians, like those of every culture, also seek advice for health-related decisions from relatives or friends, some of whom may be physicians and nurses. This form of education may supersede the advice of physicians who are known only through a transient patient-provider relationship. Thus, the casual dialogue about HBV vaccination shared by HCWs among acquaintances, friends and family, and accordingly, their willingness to advise vaccination, may affect HBV vaccination coverage among the most vulnerable population—newborns.

The purpose of this study was to identify factors associated with HCWs recommendations for newborn HBV vaccination. The hypothesis was that HCWs who were not vaccinated, and thus had no personal experience with the vaccine, would be more likely to recommend against HBV vaccination of newborns.

## Methods

### Study design and sample

A cross-sectional study on the prevalence and awareness of HBV and other infections was conducted among randomly selected physicians and nurses employed in seven Georgian hospitals between January 1, 2006 and December 31, 2007. Self-administered questionnaires included a module on recommendations for HBV vaccine for newborns. These physicians were also underwent serological testing for HBV, HCV and HIV.

Seven major hospitals located in five cities were chosen to be representative of Georgia, including three from Tbilisi, the capital and largest city, two from Western Georgia and two from Eastern Georgia. Study participants were selected using simple random sampling of physicians and nurses of selected departments based on lists provided by the hospitals of all full-time employees. The departments enlisted for the study included internal medicine, obstetrics, surgery, intensive care, dialysis and paediatrics. Enrolment continued until the designated number (proportional to number of HCWs per hospital) was met.

### Enrollment and data collection

Potential study participants were invited to voluntarily participate during a private interview with a researcher. The study, including the content of the questionnaire and testing for HBV, HCV and HIV were explained and informed consent was obtained. To maintain confidentiality, a study code was provided to each HCW and the code was used to label questionnaires and blood samples. Furthermore, a study physician provided test results to all participants along with counselling and referral when further tests were needed. Approval by both Georgian and US Ethics Committees was obtained prior to initiation of the study.

The questionnaire was pilot tested in a small group of physicians and nurses and modified prior to the study. Formative evaluation of the questionnaire was conducted using qualitative methods to assess clarity of the questions. The questionnaire included demographic, clinical and occupational characteristics and knowledge, attitudes, and practices requiring exposure to, and protection from, blood-borne pathogens. The HCWs' willingness to recommend HBV vaccine to newborns was assessed by the question: “Would you advise HBV vaccine for newborns?” Factors hypothesized to be associated with recommending HBV vaccine for newborns were included in the analyses, including self-reported history of HBV vaccination, fear of vaccine-related side effects, misconceptions of vaccine effectiveness, demographic characteristics (e.g., age, gender), clinical characteristics (e.g., immune status to HBV and HCV) and occupational characteristics (e.g., job category), position (e.g., physicians, nurses), and risky occupational behaviour (e.g., accidental needle-sticks and blood splashes).

### Laboratory analysis

Venous blood (2–3 ml) was drawn from health care workers and transported daily from the hospitals to the laboratory in Tbilisi for storage and analysis. To determine HBV infection status, third generation ELISA tests (manufacturer Orgenics, Israel) were used for HBsAg and anti-HBc. All HBsAg-positive samples were further investigated by confirmatory assays, with immunoreaction of neutralization combined with HBsAg.

### Statistical analysis

Data were checked for quality, double-entered into SPSS (Version 13.0) and verified, before analysis using SPSS and SAS (Version 9.2). Bivariate analyses were performed to assess the association between a history of being vaccinated and each potential predictive factor as well as willingness to advise vaccination for newborns. Multivariate analysis was conducted using Poisson regression with robust variance estimators to identify the variable most predictive of recommending HBV vaccine to newborns.

## Results

Of the 1600 randomly selected HCWs, 1386 (86%) enrolled in the study and 1328 (83%) provided sufficient information for inclusion in the analysis (Answered the question: Would you recommend HBV vaccine to children?). Of these 1328 HCWs, 22.80% were 35 years of age or younger; 20.86% were males. The study participants were from a broad range of specialties, including surgery (28.60%), internal medicine (19.58%), intensive care (18.45%), obstetrics-gynaecologists (15.44%), dialysis (6.93%), paediatricians (6.63%) and others (4.14%). 46.76% of respondents were physicians, 28.40% were from the age group of 35 or younger. 387 HCWs (29.14%) had HBc antibodies and 26 (1.96%) were HBsAg positive.

Overall, 478 (36%) study participants reported recommending against HBV vaccination for children. Of the 279 who reported a specific reason, the most common was concern about an adverse vaccine event (38%, 111/279) followed by belief that the vaccine was not effective (23%, 64/279).

Based on the bivariate analysis, the strongest correlate for likelihood of recommending HBV vaccination was the HCW’s own vaccination status. Unvaccinated HCWs were much more likely to recommend *against* HBV vaccine than their vaccinated colleagues (40.40% vs 11.40%, respectively, prevalence ratio (PR) = 3.54; 95% CI: 2.38, 5.29). Age was also a factor, with older HCWs more likely to recommend against HBV vaccine compared to those younger than age 35 (39.20% vs 28.40%, respectively). There was no substantive difference between the recommendations for physicians compared to nurses. A third of paediatricians recommended against HBV vaccine, similar to other HCWs. Dialysis HCWs were the most likely to recommend vaccination, with only 10.9% (PR=3.03; 95% CI: 1.58, 5.85) recommending against it. By multivariate analysis, the only significant predictor of recommending HBV vaccine for newborns was the HCW’s own vaccination status (Table 1).

**Table 1:** Bivariate and multivariate analyses from HCW survey: factors related to not recommending HBV vaccine for children, Georgia, 2006-2007

**Demographic and occupational factors**	**Total No.**	**Recommending against HBV vaccine for children No. (%)**	**Bivariate Prevalence Ratio (PR) and 95% CI**	**Adjusted Prevalence Ratio (aPR) and 95% CI**
Self reported vaccination status				
Unvaccinated	1120	453 (40.40)	1.00	1.00
Vaccinated	193	22 (11.40)	3.54 (2.38, 5.29)	1.45 (1.18,1.72)
Missing values	15			
Age group				
>35	931	365 (39.20)	1.00	1.00
<=35	303	86 (28.40)	0.72 (0.59,0.88)	0.89 (0.71,1.05)
Missing values	94			
Gender				
Female	1050	386 (36.80)	1.00	1.00
Male	277	92 (33.20)	0.90 (0.75, 1.09)	0.89 (0.72,1.09)
Missing values	1			
Position				
Physician	621	234 (37.70)	1.00	1.00
Nurse	700	243 (34.70)	0.92 (0.79,1.06)	0.95 (0.89,1.12)
Missing values	7			
Department				
Paediatrics	88	29 (33.00)	1.00	1.00
Surgery	381	133 (34.90)	1.06 (0.76,1.47)	1.08 (0.79,1.63)
Dialysis	92	10 (10.90)	3.03 (1.58,5.85)	0.83 (0.58,1.19)
ICU	245	103 (42.00)	1.27 (0.91,1.78)	1.19 (0.86,1.67)
Internal medicine	260	102 (39.20)	1.19 (0.85,1.66)	1.12 (0.81,1.54)
Obstetrician –	205	78 (38.00)	1.15 (0.82,1.63)	1.09 (0.78,1.52)
Gynecologist				
Lab	55	21(38.20)	1.16 (0.74,1.81)	1.00 (0.63,1.59)
Missing values	2			

### Factors related to vaccination status

Age group was significantly associated with vaccination status: HCWs younger than 35 were more likely to be vaccinated compared to older colleagues (22.80% vs 12.30%, PR=1.85; 95% CI:1.42, 2.43). There was no statistically significant difference in the prevalence of anti-HBc in vaccinated and unvaccinated persons (28.10% anti-HBc positives among vaccinated versus 29.40% among unvaccinated HCWs). In the subgroup of HCWs 35 years or younger, the prevalence of anti-HBc was 20% among vaccinated persons as compared to 24.20% among unvaccinated HCWs (difference not significant). Gender, occupation and history of occupational exposure to patient’s blood (blood splash, needle-stick, cut with contaminated instrument) were not associated with HBV vaccination.

## Discussion

As in other countries [10,12], concerns about vaccine safety persist in Georgia among the general population as well as among HCWs. What may be unique to this country is a pervasive and persistent concern that transcends the safety data for this vaccine. While HBV vaccine has an excellent safety and efficacy record [13-17], perceptions in Georgia have been affected by general concerns about vaccine safety and effectiveness and specifically by one highly publicized neurologic event that occurred a week after an HBV vaccination in Georgia [10]. Overcoming these misconceptions to develop a strong and sustainable universal vaccination program is crucial to reduce HBV in newborns and children and help eliminate HBV- associated liver disease and the economic burden of this disease for Georgia.

This study found that 36% of HCWs did not recommend HBV vaccination for newborns. Only two groups of HCWs appeared to be importantly different: dialysis workers who see the impact of HBV among their patients at very high risk of the infection, and HCWs who are vaccinated against HBV. However, as evidence of the intractable nature of vaccine safety concerns, one in every ten of these HCWs would also recommend against HBV vaccine for newborns. Age correlated with vaccine status, with older HCWs being the least likely to be vaccinated and least likely to recommend vaccine. These findings provide insight into the demographics of concern about vaccine safety and inform the development of potential interventions to improve knowledge about HBV vaccination.

There was no statistically significant difference in the prevalence of anti-HBc in vaccinated and unvaccinated persons. This could be probably explained by the fact that during the HCW’s HBV vaccination campaign in 2001 there was no HBV antibody testing conducted prior to HBV vaccination and those being already HBV infected without knowing it received the vaccine.

During the study period over 80% of HCWs were not yet vaccinated against HBV. While this finding is similar to many developing countries [18-20], it is the polar opposite of most developed countries where the majority of HCWs are vaccinated [21-24]. These unvaccinated HCWs are substantially less likely to recommend vaccine to newborns. Fear of adverse events and lack of knowledge regarding vaccine efficacy are major factors affecting many HCWs recommendations and personal health practices. While these issues may be addressed by educational programs, the specific experiences of this population suggest educational programs alone are insufficient. Targeted programs which specifically address the publicized event and shed light on the facts may help assuage some concerns.

Personal experience with HBV vaccine appears to be associated with recommending vaccine to newborns.

Having received HBV vaccine is associated with a healthcare worker recommending the vaccine to newborns. The reasons a healthcare worker might receive the vaccine may be complex. Yet access to the vaccine may be a key factor. If the vaccine is available to the HCW and the barrier of cost is removed, it seems likely that the HCW may consider the personal cost-benefit relationship in a different context. They ultimately may see the benefits of the vaccine as more favourable, receive the vaccine and ultimately be more likely to recommend HBV vaccination for newborns as well as others.

Vaccination programs for HCWs can be justified based on their occupational risk of infection. Cost is always a factor in any vaccine initiative. Some hospitals in a neighbouring country, Turkey, have screened HCWs upon employment and identified those susceptible to HBV for vaccination. This screening and vaccine program is cost-effective given the relatively large proportion of HCWs that have already been exposed to HBV. In Georgia, approximately 30% of HCWs have evidence of prior HBV exposure and thus would not directly benefit from the vaccine. The remaining HCWs would benefit from the vaccine and safety data suggests that those HCWs who were already vaccinated would not be harmed by revaccination (essentially an additional booster) [25,26]. This last point is important because one significant barrier to a screening program is the potential harm caused by employers identifying HCWs who are infectious. Thus, creating an accessible national program for HCW vaccination must rely on a confidential screening program, or universal vaccination for those with an unreliable vaccination history.

Cost-effectiveness studies on HBV vaccine programs for HCWs typically focus on the occupational risks of HBV infection and benefit of the vaccine to protect HCWs and patients [27,28]. This study suggests increasing the proportion of HCWs vaccinated for HBV may increase vaccination coverage of newborns and children.

This study had a strong sampling design and benefited from laboratory testing to establish exposure to HBV. Like all studies, it also had limitations. The primary limitation is that all other information was self-reported. Based on the results, there does not appear to be substantial bias due to social desirability as the proportion of HCWs recommending vaccine is relatively low. Another limitation is that participation was not 100%. It is possible that HCWs with active disease were more likely to refuse participation. However, this would not alter the overall conclusions.

The prevalence of HBV infection (anti-HBc) among study participants was about 29%, higher than the prevalence among the general population in Georgia (20%) [29]. This difference could be explained by the occupational exposure to HBV among HCWs. Even if the majority of HCWs are not likely to develop significant liver disease sequelae if infected later in life, they can transmit the virus to patients and household members who have not been vaccinated. Thus, it would be useful to assess cost-effectiveness of screening for both HBV antibodies prior to vaccinating susceptibles and universal vaccination for HCWs in the absence of screening. In either case, cost-effectiveness analyses of HBV vaccination programs for HCWs should broaden their assessment of benefits.

In 2009 the Ministry of Labour, Health, and Social Affairs of Georgia and the National Center for Diseases Control established a hepatitis B catch-up vaccination program for at-risk healthcare workers, medical students, and adolescents in three regions of the country: Tbilisi (capital city with 1/3 of Georgia population), Imereti and Adjara (regions in western Georgia) with support of Rostropovich-Vishnevskaya Foundation.

By 2010, 12,963 health care workers have been screened for hepatitis B (Anti-HBc) and 4,579 (35.3%) appeared to be Anti-HBc(+). The rest of HCWs were offered administration of HBV vaccine on a voluntary basis, from which 7,000 have been vaccinated against hepatitis B.

No study was conducted to estimate the impact of vaccination campaign among HCWs on the attitude of HCWs towards HBV vaccine and their willingness to recommend it to the children.

## Conclusion

Our study reinforced the importance of HBV vaccination among HCWs for the obvious direct occupational benefits such protection confers as well as possibly recommending vaccination for newborns. This study leads us to recommend the education and outreach targeted towards HCWs who are least likely to perceive the benefits of HBV vaccination; those over age 35, may improve the long-term HBV vaccine prevalence rates. Such programs must also remove barriers to vaccination such as concern for social consequences and personal financial burden.

## Competing interests

The authors declare that they have no competing interests.

## Authors’ contributions

MB – PI, development of study design, analysis of data, and manuscript writing. GK – Manager, overall management of the study and participation in manuscript writing. MT – Epidemiologist, participation in data collection and analysis. DM- Participation in development of the study design and editing the manuscript. WT- Participation in development of research tools and editing the manuscript. JD- Participation in drafting and editing the manuscript. KN- editing the manuscript. LAM – US Co-PI, collaborative overall supervision of the study, manuscript writing. All authors read and approved the final manuscript.

## Pre-publication history

The pre-publication history for this paper can be accessed here:

http://www.biomedcentral.com/1471-2334/12/362/prepub

## References

[B1] Butsashvili M Tsertsvadze T McNutt LA Prevalence of hepatitis B, hepatitis C, syphilis and HIV in Georgian blood donors Eur J Epidemiol 2001 17 7 693 695 10.1023/A:1015566132757 12086085

[B2] Iashina TL Favorov MO Shakhgil'dian IV Firsova SN Eraliev AE Zhukova LD Reznichenko RG The prevalence of the markers of viral hepatitis B and delta among the population in regions differing in the level of morbidity Vopr Virusol 1992 37 4 194 196 1471341

[B3] Nurgalieva ZZ Hollinger FB Graham DY Zhangabylova S Zhangabylov A Epidemiology and transmission of hepatitis B and C viruses in Kazakhstan World J Gastroenterol 2007 13 8 1204 1207 17451200 10.3748/wjg.v13.i8.1204 PMC4146994

[B4] Zacharakis G Koskinas J Kotsiou S Pouliou E Papoutselis M Tzara F Vafeiadis N Maltezos E Archimandritis A Papoutselis K Natural history of chronic hepatitis B virus infection in children of different ethnic origins: a cohort study with up to 12 years' follow-up in northern Greece J Pediatr Gastroenterol Nutr 2007 44 1 84 91 10.1097/01.mpg.0000243438.47334.07 17204959

[B5] Slowik MK Jhaveri R Hepatitis B and C viruses in infants and young children Semin Pediatr Infect Dis 2005 16 4 296 305 10.1053/j.spid.2005.06.009 16210109

[B6] Lewis E Shinefield HR Woodruff BA Safety of neonatal hepatitis B vaccine administration Pediatr Infect Dis J 2001 20 1049 1054 10.1097/00006454-200111000-00009 11734710

[B7] Tovo PA Lazier L Versace A Hepatitis B virus and hepatitis C virus infections in children Curr Opin Infect Dis 2005 18 3 261 266 10.1097/01.qco.0000168388.24142.2b 15864105

[B8] Van Herck K Van Damme P Benefits of early hepatitis B immunization programs for newborns and infants Pediatr Infect Dis J 2008 27 10 861 869 10.1097/INF.0b013e318173966f 18776823

[B9] Papaevangelou V Hadjichristodoulou C Cassimos DC Pantelaki K Tzivaras A Hatzimichael A Theodoridou M Seroepidemiology of hepatitis B in Greek children 6 years after the implementation of universal vaccination Infection 2008 36 2 135 139 10.1007/s15010-007-7096-6 18231718

[B10] Case report: encephalomyelitis caused by hepatitis B vaccination 2007 Available at: http://www.vaclib.org/basic/Case_Report.doc. Accessed: April 29

[B11] Topuridze M ButsaShvili M Kamkamidze G Hepatitis B vaccine coverage among health care workers: barriers to coverage Infect Control Hosp Epidemiology 2010 31 2 158 164 10.1086/649795 20038247

[B12] Balinska MA Hepatitis B vaccination and French Society ten years after the suspension of the vaccination campaign: how should we raise infant immunization coverage rates? Review J Clin Virol 2009 46 3 202 205 Review 10.1016/j.jcv.2009.07.024 19716764

[B13] Smith PJ Kennedy AM Wooten K Gust DA Pickering LK Association between health care providers' influence on parents who have concerns about vaccine safety and vaccination coverage Pediatrics 2006 118 5 e1287 e1292 10.1542/peds.2006-0923 17079529

[B14] Alavian SM Mansouri S Abouzari M Assari S Bonab MS Miri SM Long-term efficacy of hepatitis B vaccination in healthcare workers of Oil company hospital, Tehran, Iran (1989–2005) Eur J Gastroenterol Hepatol 2008 20 2 131 134 10.1097/MEG.0b013e3282f1cc28 18188034

[B15] Szmuness W Stevens CE Harley EJ Zang EA Alter HJ Taylor PE Hepatitis B vaccine in medical staff of hemodialysis units: efficacy and subtype cross-protection N Engl J Med 1982 307 1481 1486 10.1056/NEJM198212093072403 6755247

[B16] Mac Mahon BJ Helminiak C Wainwright RB Bulkow L Wainwright K Frequency of adverse reactions to hepatitis B vaccine in 43,618 persons Am J Med 1992 92 254 256 10.1016/0002-9343(92)90073-K 1532114

[B17] Zanetti AR Update on hepatitis B vaccination in Italy 10 years after its implementation Vaccine 2001 19 2380 2383 10.1016/S0264-410X(00)00458-8 11257364

[B18] Braka F Nanyunja M Makumbi I Mbabazi W Kasasa S Lewis RF Hepatitis B infection among health workers in Uganda: evidence of the need for health worker protection Vaccine 2006 24 47–48 6930 6937 17027122 10.1016/j.vaccine.2006.08.029

[B19] Suckling RM Taegtmeyer M Nguku PM Al-Abri SS Kibaru J Chakaya JM Tukei PM Gilks CF Susceptibility of healthcare workers in Kenya to hepatitis B: new strategies for facilitating vaccination uptake J Hosp Infect 2006 64 3 271 277 10.1016/j.jhin.2006.06.024 16926061

[B20] Talaat M Kandeel A El-Shoubary W Bodenschatz C Khairy I Oun S Mahoney FJ Occupational exposure to needlestick injuries and hepatitis B vaccination coverage among health care workers in Egypt Am J Infect Control 2003 31 8 469 474 10.1016/j.ajic.2003.03.003 14647109

[B21] Simard EP Miller JT George PA Wasley A Alter MJ Bell BP Finelli L Hepatitis B vaccination coverage levels among healthcare workers in the United States, 2002–2003 Infect Control Hosp Epidemiol 2007 28 7 783 790 10.1086/518730 17564979

[B22] Batista SMF Andreas MSA Borges AMT Lindenberg ASC Silva AL Fernandes TD Pereira EF Basmage EAM Cardoso DDP Seropositivity for hepatitis B virus, vaccination coverage, and vaccine response in dentists from Campo Grande, Mato Grosso do Sul, Brazil Mem Inst Oswaldo Cruz 2006 101 3 263 267 10.1590/S0074-02762006000300006 16862319

[B23] Dannetun E Tegnell A Torner A Giesecke J Coverage of hepatitis B vaccination in Swedish healthcare workers J Hosp Infect 2006 63 2 201 204 10.1016/j.jhin.2006.01.014 16621139

[B24] Vranckx R Jacque P De Schrijver A Moens G Hepatitis B vaccination coverage in Belgian health care workers Infection 2004 32 5 278 281 10.1007/s15010-004-2204-3 15624891

[B25] Chen W Gluud C Vaccines for preventing hepatitis B in health-care workers Cochrane Database Syst Rev 2005 19 4 CD000100 16235273 10.1002/14651858.CD000100.pub3

[B26] Floreani A Baldo V Cristofoletti M Renzulli G Valeri A Zanetti C Trivello R Long term persistence of anti-HBs after vaccination against HBV: an 18 year experience in health care workers Vaccine 2004 22 5–6 607 610 14741151 10.1016/j.vaccine.2003.09.001

[B27] Kuruuzum Z Yapar N Avkan-Oguz V Aslan H Ozbek OA Cakir N Yuce A Risk of infection in health care workers following occupational exposure to a noninfectious or unknown source Am J Infect Control 2008 36 10 e27 e31 10.1016/j.ajic.2008.05.012 19084160

[B28] Tu HA Woerdenbag HJ Kane S Riewpaiboon A van Hulst M Postma M Economic evaluations of hepatitis B vaccination for developing countries Expert Rev Vaccines 2009 8 7 907 920 10.1586/erv.09.53 19538116

[B29] National Center Of Disease Control And Public Health (Ncdc), Bulletin http://www.ncdc.ge/GEO/Publications/Bulletin/ bulletin_2005/Bul_1.pdf

